# Ursodeoxycholic Acid Influences the Expression of p27^kip1^ but Not FoxO1 in Patients with Non-Cirrhotic Primary Biliary Cirrhosis

**DOI:** 10.1155/2014/921285

**Published:** 2014-02-05

**Authors:** Malgorzata Milkiewicz, Justyna Kopycińska, Agnieszka Kempińska-Podhorodecka, Tara Haas, Dimitrios P. Bogdanos, Elwyn Elias, Piotr Milkiewicz

**Affiliations:** ^1^Medical Biology Laboratory, Pomeranian Medical University, Aleja Powstancow Wlkp. 72, 70-111 Szczecin, Poland; ^2^Angiogenesis Research Group, Faculty of Health, York University, Toronto, Canada; ^3^Division of Transplantation Immunology and Mucosal Biology, Institute of Liver Studies, King's College London Medical School at King's College London Hospital, London SE5 9RS, UK; ^4^Liver Unit, University of Birmingham, Birmingham B15 2TH, UK; ^5^Liver Research Laboratories, Pomeranian Medical University, 70-111 Szczecin, Poland; ^6^Department of General, Transplant and Liver Surgery, Liver and Internal Medicine Unit, Warsaw Medical University, 02-092 Warsaw, Poland

## Abstract

*Background*. Enhanced expression of cell cycle inhibitor p27^kip1^ suppresses cell proliferation. Ursodeoxycholic acid (UDCA) delays progression of primary biliary cirrhosis (PBC) but its effect on p27^kip1^ expression is uncertain. *Aims*. To analyze the expression of p27^kip1^ and its transcription modulator FoxO1 in patients with PBC, and to assess the impact of UDCA on this pathway. *Materials and Methods*. The examined human tissue included explanted livers from patients with cirrhotic PBC (*n* = 23), primary sclerosing cholangitis (PSC; *n* = 9), alcoholic liver disease (ALD; *n* = 9), and routine liver biopsies from patients with non-cirrhotic PBC (*n* = 26). Healthy liver samples served as controls (*n* = 19). Livers of FoxO-deficient mice were also studied. mRNA and protein expressions were analyzed by real-time PCR and Western blot. *Results*. p27^kip1^ expression was increased in cirrhotic and non-cirrhotic PBC. FoxO1 mRNA levels were increased in PBC (8.5-fold increase versus controls). FoxO1 protein expression in PBC was comparable to controls, but it was decreased in patients with PSC and ALD (63% and 70% reduction, respectively; both *P* < 0.05 versus control). UDCA-treated non-cirrhotic patients with PBC showed decreased expression of p27^kip1^ mRNA. *Conclusion*. PBC progression is characterized by a FoxO1-independent increase of p27^kip1^ expression. In early PBC, UDCA may enhance liver regeneration via p27^kip1^-dependent mechanism.

## 1. Introduction

Primary biliary cirrhosis (PBC) is a chronic cholestatic liver disease characterized by an autoimmune-mediated inflammatory destruction of biliary epithelial cells [1–4] of the small intrahepatic small bile ducts. The standard care in PBC consists of the oral administration of ursodeoxycholic acid (UDCA) [5]. UDCA has been shown to improve clinical and biochemical indices in a variety of liver diseases, but the precise mechanism by which UDCA improves liver function remains unclear. Nevertheless, UCDA regulates the endogenous secretion of bile acid, reduces the cytotoxicity of endogenous bile acids, modulates the production of inflammatory cytokine, is protective against oxidative stress, and inhibits cell apoptosis [6, 7]. Recent reports have denoted another important role of UDCA as a key modulator of cell cycle [8].

The liver possesses a notable capacity to regenerate after partial resection until it achieves its primary size. Contrary to most cell types, mature hepatocytes preserve significant proliferative potential even after terminal differentiation. Recent data have convincingly demonstrated that the cell cycle regulator p27^kip1^ contributes to the inhibition of proliferative activity of differentiated hepatocytes. p27^kip1^ is a member of Cip/Kip family of broad spectrum cyclin dependent kinase (Cdk) inhibitors which suppress cell cycle progression in different cell types by negative regulation of Cdks. p27^kip1^ appears to regulate the G1/S transition either by the prevention of Cdk-dependent phosphorylation of pRb [9, 10] or by inhibition of activity of E-Cdk2 and A-Cdk2 cyclin complexes in mature nonproliferative hepatocytes [11]. During the development of rat liver, p27^kip1^ is strongly expressed in fetal liver and declines afterwards. However, relatively moderate levels of p27 can be detected in differentiated quiescent hepatocytes. The increased number of hepatocytes noted in p27 knockout mice further underlines the important role of this gene in liver regeneration and makes it an appealing target in liver research [12].

The liver plays a crucial role in adaptation to stress. One of the most important stress response pathways is regulated by FoxO transcription factors. The FoxO subfamily composes of 4 members (FoxO1, FoxO3a, FoxO4, and FoxO6) and is characterized by the forkhead DNA-binding domain and broad physiological functions [13, 14]. In response to a variety of external stimuli, FoxOs orchestrate gene expressions involved in oxidative-stress resistance, DNA repair, and glucose metabolism and function to prevent cellular proliferation [15]. A FoxO subfamily of transcription factors appears to be important as positive regulator of p27^kip1^ expression [14, 16]. Although the role of FoxO transcription factors as critical modulators of the cell cycle homeostasis has been previously demonstrated in different cell types, the study of its expression in liver tissue has been limited so far to research carried out on cultured hepatocytes [17] or stellate cells [18].

We hypothesized that an activated FoxO1/ p27^kip1^ signaling axis can hinder the efficacy of cellular regeneration induced by early pathological changes specific for PBC. Knowing that hepatocytes are able to arrest reversibly a cell growth, we focused on the genes that repeatedly correlate with modulation of the cell cycle. To the best of our knowledge, this is the first study to investigate in great detail the expression of FoxO1 and p27^kip1^ in human liver tissue of *patients with non-cirrhotic and cirrhotic PBC.* Finally, the interrelationship between FoxO1 and its possible downstream target p27^kip1^ was investigated in the livers of mice harboring a conditional deletion of FoxO1/3a/4.

## 2. Materials and Methods

### 2.1. Liver Tissue

Human liver tissue specimen from non-cirrhotic and cirrhotic liver disease patients was analyzed. The cirrhotic groups included 23 patients with PBC, 9 with primary sclerosing cholangitis (PSC) and 9 with alcoholic liver disease (ALD). A fourth group of non-cirrhotic PBC (*n* = 26) was also studied. Liver tissue specimens were obtained from non-cirrhotic PBC when they underwent routine percutaneous liver biopsies for histological assessment of the stage of the disease and fibrosis scoring. The cirrhotic groups liver tissue specimens were obtained from explanted livers of patients with PBC, PSC, and ALD who underwent liver transplantation.

Amongst the 26 non-cirrhotic PBC patients, 10 were receiving ursodeoxycholic acid (UDCA) in the dose 13–15 mg/kg b.w. before obtaining liver tissue, while 16 were UDCA-naive. Samples (*n* = 19) from liver tissues with no macroscopic changes obtained during large margin resections of hepatocellular carcinoma served as controls. Patients and controls were matched for age and sex and an informed consent was obtained from each patient. The study protocol was approved by the ethics committee of Pomeranian Medical University and conformed to the ethical guidelines of the 1975 Declaration of Helsinki.  Table 1 summarizes clinical and laboratory features of the study participants.

### 2.2. Human Liver Tissue Preparation

Liver tissue (~1 cm^3^; from controls, ALD, PSC, and cirrhotic PBC) was immediately frozen in liquid nitrogen and stored at −75°C until used. Tissue specimens obtained by percutaneous needle liver biopsy (non-cirrhotic PBC) were cut into two pieces. One part (2-3 mm^2^) was stored in RNA *later* (AM7021; Applied Biosystems, Carlsbad, CA, USA) and the second one was fixed in 10% neutral-buffered formalin and subsequently embedded in paraffin for histological assessment. Serial sections (5 *μ*m) were stained with hematoxylin and eosin. The liver tissue from non-cirrhotic PBC patients was analyzed by a pathologist blinded to the clinical and laboratory features of the included subjects (Table 2).

### 2.3. Animal Study: Generation of Mx-Cre^+^: FoxO1/3/4^L/L^ Mice

All experimental procedures that involved animals were approved by the York University Animal Care Committee. Mice harboring the interferon-inducible transgene Mx-Cre in a FoxO1/3/4^L/L^ background were generated as previously described [19]. Cre expression and subsequent FoxO1/3/4 excision was induced in 4-5-week-old mice by three intraperitoneal injections of 300 *μ*g polyinosinepolycytidylic (pIpC, Sigma-Aldrich) administered every other day over 5 days. pIpC was also administered to Mx-Cre^−^ littermate controls. All mice were housed and maintained in a pathogen-free animal facility in microisolator cages. Animals were monitored daily and were sacrificed at designated times after induction with pIpC. Murine livers extraction was performed under aseptic conditions and isoflurane anesthesia, at minimum of 2 weeks following the final injection of pIpC. Total RNA and proteins were extracted directly from the fresh tissue.

### 2.4. RNA Extraction and cDNA Synthesis

Total RNA from human and murine liver tissues was extracted using the RNeasy Mini kit (Qiagen, Valencia, USA), according to the manufacturer's protocol. The isolated RNA was stored at −75°C until used. cDNA synthesis was carried out using Superscript II RT kit (Invitrogen, Carlsbad, CA, USA) according to the protocol previously described [20]. Newly synthetised cDNA was stored at −20°C.

### 2.5. Quantification of Gene Expression Using Real-Time PCR

The expression of specific target genes was measured by quantitative real-time PCR using commercially available Gene Expression Assays and 7500 Fast Real-Time PCR System (Applied Biosystems). The following assays were used in the study: human FoxO1 (Hs00231106_m1); human p27^kip1^ (Hs00153277_m1); murine FoxO1 (Mm00490672_m1); murine p27^kip1^ (Mm00438168_m1), and control human GAPDH (Hs99999905_m1) and murine GAPDH (Mm99999915_g1). A 20 *μ*L reaction mixture contained 10 *μ*L of TaqMan Gene Expression PCR Master Mix (Applied Biosystems, Foster City, CA, USA), 2 *μ*L diluted cDNA template, and 1 *μ*L of the probe/primer mix. The fluorescence data were analyzed with 7500 Software v2.0.2. (Applied Biosystems, Carlsbad, CA, USA). The expression of target genes was calculated using the ΔΔCt method of relative quantification.

### 2.6. Protein Expression Analysis

Proteins from human (control *n* = 19; ALD *n* = 9; PSC *n* = 9; and cirrhotic PBC *n* = 23) and murine liver tissue were extracted through homogenization in an ice-cold RIPA buffer (50 mM Tris-HCl pH = 8, 150 mM NaCl, 1% NP-40, 0.5% NaDOC, 0.1% SDS, 1 mM EDTA, 100 mM PMSF, and 100 mM NaF) containing protease inhibitor cocktail and PhosSTOP (Roche Diagnostics GmbH, Mannheim, Germany). Protein quantification was made using the bicinchoninic acid assay (Micro BCA Protein Assay Kit; Thermo Scientific, Waltham, MA, USA). 80 *μ*g of total protein extracts from each liver sample was electrophoresed in SDS polyacrylamide gels. Subsequently the gels were blotted into PVDF membranes (Thermo Scientific, Rockford, IL, USA) under semidry transfer conditions. Membranes were blocked overnight (4°C) with TBST containing 5% (w/v) milk (Merck) and then probed using the following primary antibodies: anti-FoxO1 (9454; Cell Signaling, 1 : 500 dilution), anti-p27^kip1^ (sc-1641; Santa Cruz, 1 : 500), anti-*α*/*β*-tubulin (2148, Cell Signaling, 1 : 1000 dilution), and anti-*β*-actin (sc-47778; Santa Cruz, 1 : 1000 dilution). For the detection of antigen-antibody complexes, peroxidase conjugated anti-rabbit secondary antibody (NA9340V, Amersham, GE Healthcare, UK; 1 : 5000 dilution) or anti-mouse secondary antibody (NA9310V, Amersham; 1 : 5000 dilution) was used. Protein expression was detected using an enhanced chemiluminescence detection system (Chemiluminescent HRP Substrate, Millipore, Billerica, MA, USA) and visualized on autoradiographic films (Amersham Hyperfilm ECL). Band densities were quantified by densitometry (GeneSnap ver. 7.01 software; Syngene, Cambridge, UK) after normalization to *α*/*β*-tubulin or *β*-actin.

### 2.7. Statistical Analysis

Statistical analysis was performed with ANOVA and Fisher's PSLD using StatView software version 5.0 (SAS Institute Inc. Cary, NC, USA). Data were expressed as mean ± SE for at least nine separate experiments. Results were considered statistically significant when two-side  *P* values were less than 0.05.

## 3. Results 

### 3.1. Expression of FoxO1

**Figure 1:**
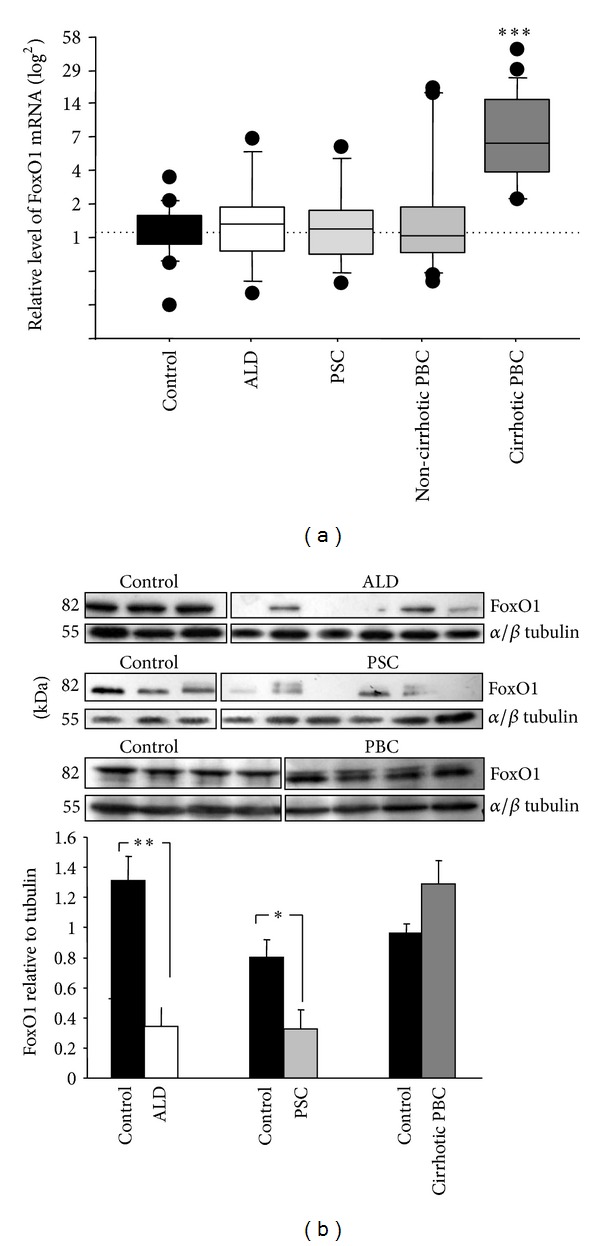
FoxO1 expression in non-cirrhotic PBC, cirrhotic PBC, ALD, PSC, and controls. (a) mRNA and (b) protein. Levels of gene expression presented as fold-change relative to control were normalized with glyceraldehydes 3-phosphate dehydrogenase (GAPDH). Changes in FoxO1 protein levels were determined by densitometry analyses after normalization to *α*/*β* tubulin as a control for loading. Bars indicate the mean ± SEM (*P < 0.05; **P < 0.005; ***P < 0.0001 versus control).

The qPCR analysis of human liver tissue showed a notable upregulation of FoxO1 mRNA in cirrhotic liver tissue of patients with PBC compared to controls (8.5-fold increase; *P* < 0.0001). The levels of FoxO1 mRNA in PSC and ALD patients were comparable to those of controls (Figure 1(a)). Western blot analysis revealed no statistically significant difference in FoxO1 protein levels between cirrhotic patients with PBC and controls. Interestingly, the levels of FoxO1 protein were decreased in PSC (2.5-fold decrease versus control; *P* < 0.05) and in ALD (3.7-fold decrease versus control; *P* < 0.005) (Figure 1(b)).

### 3.2. Expression of p27^kip1^

**Figure 2:**
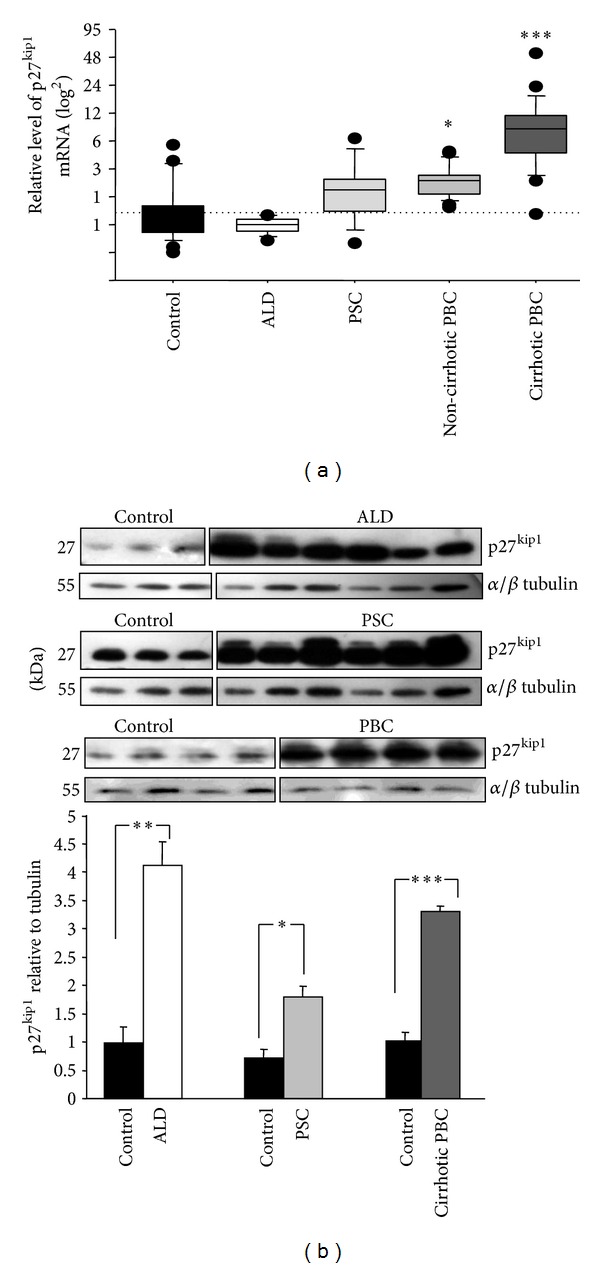
p27^kip1^ expression in non-cirrhotic PBC, cirrhiotic PBC, ALD, PSC, and controls. (a) mRNA and (b) protein. Levels of gene expression presented as fold-change relative to control were normalized with GAPDH. Changes in p27^kip1^ protein levels were determined by densitometry analyses after normalization to *α*/*β* tubulin as a control for loading. Bars indicate the mean ± SEM (*P < 0.05; **P < 0.005; ***P < 0.0001 versus control).

p27^kip1^ was previously described as the downstream target of FoxO1. Therefore, p27^kip1^ mRNA and protein levels were also examined. The results of quantitative PCR showed a significant increase of p27^kip1^ mRNA levels in non-cirrhotic and cirrhotic patients with PBC compared to controls (2.1 ± 0.2 versus 1.3 ± 0.3, *P* = 0.04 and 9.3 ± 2.3 versus 1.3 ± 0.3, *P* = 0.0001, resp.) (Figure 2(a)). p27^kip1^ mRNA levels did not correlate with stages of fibrosis.

The levels of p27^kip1^ mRNA in ALD and PSC were comparable to control values (Figure 2(a)). Western blot analyses of tested liver specimens showed statistically significant upregulation of p27^kip1^ protein levels in cirrhotic PBC, PSC, and ALD (3.6-fold, 2-fold, and 4.5-fold increase, resp.; *P* < 0.0001, *P* < 0.05, and *P* < 0.005 versus controls, resp.) (Figure 2(b)).

### 3.3. Expression of FoxO1 and p27^kip1^ in Mx-Cre^+/−^: FoxO1/3/4 Mice

**Figure 3:**
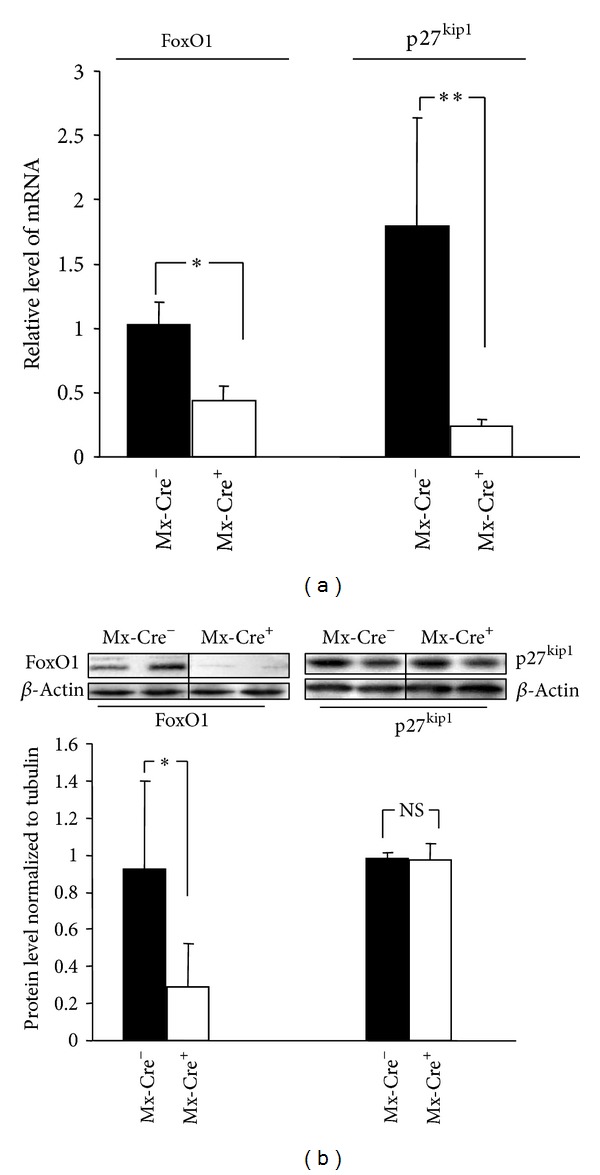
Functional studies checking the relationship between FoxO1 and p27^kip1^ in liver tissue. (a) Pharmacologically induced inactivation of FoxO1 results in suppression of FoxO1 and p27^kip1^ gene expression in the experimental Mx-Cre^+^ FoxO1/3/4 mice in comparison to control Mx-Cre^−^ FoxO1/3/4 mice. (b) FoxO1 protein expression is strongly reduced. Somatic deletion of FoxO1 gene did not affect the p27^kip1^ protein level. Results are representative of 5-6 independent experiments. Changes in protein levels were determined by densitometry analyses after normalization to *β*-actin as a control for loading. Bars indicate the mean ± SEM (*P < 0.05; **P < 0.01 versus control).

Pharmacologically induced deletion of FoxO1/3/4 genes resulted in marked decrease of FoxO1 expression on both mRNA (2.4-fold decrease; *P* < 0.05) and protein levels (3.2-fold decrease; *P* < 0.05) in Mx-Cre^+^: FoxO1/3/4 mice when compared to the control Mx-Cre^−^: FoxO1/3/4 mice group. Furthermore, a significant decrease in the expression of a downstream gene p27^kip1^ was observed at mRNA level (7.5-fold decrease at Mx-Cre^+^ versus Mx-Cre^−^ mice, *P* < 0.001). No significant changes of p27^kip1^ protein levels were noted in liver tissues of Mx-Cre^+^: FoxO1/3/4 mice compared to Mx-Cre^−^ (Figures 3(a) and 3(b)).

### 3.4. Expression of FoxO1 and p27^kip1^ in Patients Treated with UDCA and UDCA Naive

**Figure 4:**
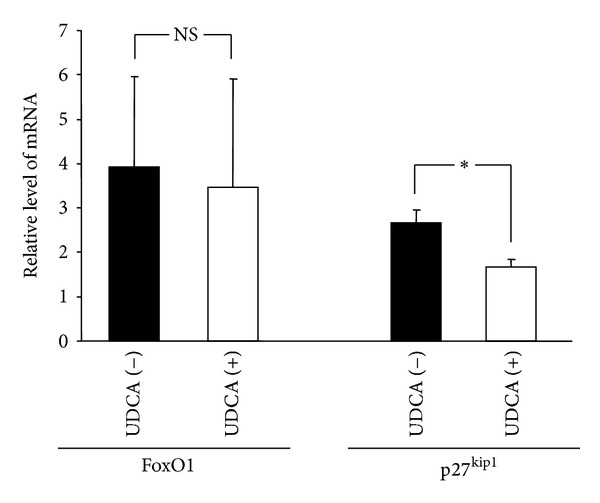
Expression of FoxO1 and p27^kip1^ mRNAs in patients with non-cirrhotic PBC who were and were not treated with UDCA (13–15 mg/kg/d). Bars indicate the mean ± SEM (*P* < 0.05; NS: non significant).

UDCA treatment of non-cirrhotic PBC patients did not affect FoxO1 mRNA level but the p27^kip1^ mRNA levels were significantly lower in UDCA-treated versus UDCA naive non-cirrhotic PBC patients (1.4 ± 0.2 versus 2.3 ± 0.3 UDCA naive; *P* = 0.036) (Figure 4). In patients with PSC, UDCA treatment did not affect the mRNA and protein levels of p27^kip1^ or FoxO1 (data not shown).

## 4. Discussion 

In this study, we demonstrated an enhanced expression of p27^kip1^ mRNA in non-cirrhotic and cirrhotic PBC livers and noted that the level of p27^kip1^ transcript was much higher in cirrhotic versus non-cirrhotic patients with PBC. The FoxO1 mRNA expression was also significantly increased in cirrhotic PBC. Intriguingly, these changes were specific for PBC as they were not observed in cirrhotic livers from patients with ALD, as well as PSC, another autoimmune cholestatic liver disease. These data led us to speculate that an increased expression of FoxO1 transcription factor with an increased level of cell cycle inhibitor p27^kip1^ may significantly contribute towards suppression of hepatocyte proliferation. Consequently, this may result in insufficient liver regeneration. Our study has also convincingly shown that UDCA administration at early stages of PBC significantly attenuates p27^kip1^ expression in human liver. However, FoxO1 levels remained unaffected.

Our data suggesting a PBC-specific increase of FoxO1 mRNA levels in cirrhotic livers is intriguing. The interpretation of this finding needs to be treated with caution, as it is well known that the synthesis of functional FoxO1 protein is controlled by multiple posttranslational modifications [21]. A thorough assessment of many upstream events that affect the amount of this protein in liver diseases is warranted to better understand the differentiation seen in cirrhotic livers from PBC compared to PSC and ALD. Nevertheless, it could be speculated that one of the potential mechanisms that could account for these findings may be disease-specific downregulation of FoxO1 transcript stability by miRNAs [22, 23].

The role of FoxO1 as the upstream modulator of p27^kip1^ gene was shown in various cell lines including murine lymphocytes, bone marrow cells, and human peripheral blood eosinophils [14, 16, 24]. Recently, the importance of FoxO1 in transcription regulation of p27^kip1^ expression was confirmed in cultured hepatic stellate cells (HSC) [25]. The published data have suggested a potential role of FoxO1 in hepatic fibrosis, as a constitutive activation of FoxO1 *in vitro* accompanied by the enhanced expression of p27^kip1^ has been demonstrated. The result of this led to the suppression of HSC proliferation, while the dominant negative form of FoxO1 and downregulation of p27^kip1^ had an opposite effect [25]. As this single study was focused on the investigation of cultured HSC, it will be challenging to draw generic conclusions applied to *in vivo* conditions of unfractioned liver cells containing less than 5% HSCs.

A negative regulator of the transition from G1 to S phase (i.e., p27^kip1^ protein) was shown to be abnormally expressed in dysplasia associated with Barrett's esophagus, sporadic colorectal adenomas, and inflammatory bowel disease-associated neoplasia [26]. Significantly lower p27 expression is commonly associated with a worse prognosis. On the other hand, the overexpression of p27^kip1^ in glioblastoma cell lines induced cell cycle arrest [27]. Also, the studies of p27^kip1^ expression in human liver were mostly done in the context of its prognostic significance in intrahepatic cholangiocarcinoma [28]. Recent liver regeneration studies indicate that stimulation in hepatocyte S-phase progression by adenovirus delivery of FoxM factor which was associated with diminished p27^kip1^ protein level was sufficient to reestablish proliferation of regenerating hepatocytes [29]. To the best of our knowledge there is no report on the expression of this cell inhibitor in autoimmune cholestatic liver diseases. The current study investigated p27^kip1^ expression in different stages of PBC. The advanced stage of the disease was accompanied by the highest p27^kip1^ mRNA and protein levels. It must be emphasized though that amongst non-cirrhotic patients with PBC p27^kip1^ increase was independent of the degree of fibrosis.

While cirrhotic PSC and ALD liver tissues did not show changes in p27^kip1^ mRNA levels, a significant increase in p27^kip1^ protein levels was noted in cirrhotic liver tissues independently of the origin of the underlying liver disease. These data could suggest that progression to advanced stages relates to an abrogation of cellular proliferation. Particularly for the liver diseases studies, it could be argued that PBC, PSC, and ALD are characterized by a decreased capacity of cellular regeneration, which could be caused by high concentration of cell cycle inhibitor p27^kip1^ in liver cells.

In order to confirm previous results suggesting that expression of p27^kip1^ is regulated by FoxO1 transcription factor, we utilized a model of transgenic mice. Inducible somatic deletion of FoxO genes in mice revealed that there is no direct effect of FoxO1 on p27^kip1^ expression in murine liver tissue. Jointly, our human and animal studies indicate that in PBC, PSC, and ALD the upregulated expression of p27^kip1^ may not be dependent on its upstream activator FoxO1.

UDCA administration was reported to exert antiproliferative effects in the large intestine and colon carcinoma in patients with PBC or PSC [30, 31]; however, the mechanisms orchestrating these phenomena are not completely understood. In our study, the effect of UDCA treatment on FoxO1 or p27^kip1^ expression was analyzed in the non-cirrhotic PBC patients. The analyses showed that transcript level of p27^kip1^ was significantly reduced in response to UDCA treatment, further suggesting its potential effect on liver regeneration. Short-lived pharmacological agents that restrain p27^kip1^ function could hypothetically be of use, as potential modality facilitating liver response to pathological stimuli.

In the animal model of colitis-associated colonic adenocarcinomas the anticarcinogenic effects of UDCA *in vivo* were demonstrated [8]. Loddenkemper et al. showed that UDCA treatment reversed the inflammation-related suppression of the cyclin dependent kinase inhibitor p27. However, the expression of p27 and the effects of UDCA in the dysplastic areas were not consistent. The apparent lack of consistency of these data with our results can be explained by the fact that the two sets of experiments were performed in different settings. Thus, reported data were obtained in an animal model of DSS-induced colon carcinomas, and it could be argued that the mechanism of UDCA effect on p27 expression may be quite distinct from that exerted in relation to our studies. Moreover, the evaluation of p27 level was based on immunohistological analysis which is only semiquantitative tool and the lack of mRNA and protein evaluations of p27 in that experimental setting do not permit for an in-depth conclusion. Of relevance to the current study, it is notable that, while cell cycle inhibitor p27 is expressed in many cell types, its unique role in cholestatic liver diseases has not been elucidated. UDCA regulates many physiological processes in a highly context-specific manner. In the context of UDCA effect on p27 expression, direct comparisons between animal carcinoma/inflamed region and liver tissue from PBC patients may be misrepresentative. Therefore, there is a need for a direct analysis of the therapeutic impact of UDCA in particular disease settings. Such analyses can provide a better understanding of the observed phenomena.

The present study indicated that p27^kip1^ may play an important role in inhibition of cell cycle during the progression of disease and that the expression of this cell cycle inhibitor is most likely FoxO1-independent. The FoxO1-p27^Kip1^ pathway was identified as a crucial regulator of the progression of cell cycle; however, the FoxO1 is also an autonomously important factor either for differentiation or maintaining differentiated cells in a nonproliferative state even in the absence of p27 [32, 33]. Based on our data, in which we observed that FoxO1 expression was comparable amongst UDCA-treated and naive subjects, we suggested that UDCA affects the p27 expression independently of the FoxO1 transcription factor. This is a potentially interesting phenomenon and needs to be investigated further. Although the results of this work are pretty consistent, an increase of the number of samples tested from patients with ALD and PSC groups could strengthen the current results.

Taken these findings together, we suggest that increased expression of p27^kip1^ may be a factor that suppresses regenerative proliferation. The observed attenuation of p27^kip1^ expression in UDCA-treated patients with early PBC may exert a positive effect on liver regeneration due to enhanced cell proliferation, but this remains to be seen in future studies.

## Figures and Tables

**Table 1:** Clinical and laboratory data on analyzed patients. *P* values between non-cirrhotic and cirrhotic patients with PBC.

	PBC non-cirrhotic *n* = 26	PBC *n* = 23	PSC *n* = 9	ALD *n* = 9
Gender (M/F)	0/26	5/18*	6/3	7/2
Age (years)	54 ± 2	56 ± 2^NS^	48 ± 5	54 ± 2
Bilirubin tot. (*μ*mol/L)	21 ± 6	114 ± 23**	133 ± 34	67 ± 32
ALP (U/L)	251 ± 36	644 ± 83**	541 ± 88	275 ± 38
AST (U/L)	63 ± 11	175 ± 28**	204 ± 42	216 ± 138
UDCA (Y/N)	10/16	23/0**	4/5	0/9

Data shown are mean ± SE.

NS: not significant; *P < 0.05; **P < 0.01 versus non-cirrhotic.

**Table 2:** Clinical data on non-cirrhotic patients with PBC who were and were not treated with UDCA.

	UDCA (+)	UDCA (−)	*P* value
Gender (M/F)	0/10	0/16	NS
Age (years)	60 ± 2	51 ± 3	NS
Total bilirubin (*μ*mol/L)	22 ± 5	21 ± 9	NS
ALP (U/L)	292 ± 76	228 ± 38	NS
AST (U/L)	87 ± 24	51 ± 11	NS
Degree of fibrosis 0-1/2-3	3/7	11/5	0.027

Data shown are mean ±SE; NS: not statistically significant; *P* > 0.05.

## Abbreviations

ALD: Alcoholic liver disease
PBC: Primary biliary cirrhosis
PSC: Primary sclerosing cholangitis
UDCA: Ursodeoxycholic acid.
